# C-reactive protein is essential for innate resistance to pneumococcal infection

**DOI:** 10.1111/imm.12266

**Published:** 2014-06-10

**Authors:** J Paul Simons, Jutta M Loeffler, Raya Al-Shawi, Stephan Ellmerich, Winston L Hutchinson, Glenys A Tennent, Aviva Petrie, John G Raynes, J Brian de Souza, Rachel A Lawrence, Kevin D Read, Mark B Pepys

**Affiliations:** 1 Wolfson Drug Discovery Unit, Centre for Amyloidosis and Acute Phase Proteins, University College LondonLondon, UK; 2 Biostatistics Unit, UCL Eastman Dental InstituteLondon, UK; 3 Department of Immunology and Infection, London School of Hygiene and Tropical MedicineLondon, UK; 4 Department of Comparative Biomedical Sciences, Royal Veterinary CollegeLondon, UK; 5 Drug Discovery Unit, Division of Biological Chemistry and Drug Discovery, University of DundeeDundee, UK

**Keywords:** anti-nuclear antibodies, C-reactive protein, host resistance, mouse knockout, pneumococcal infection

## Abstract

No deficiency of human C-reactive protein (CRP), or even structural polymorphism of the protein, has yet been reported so its physiological role is not known. Here we show for the first time that CRP-deficient mice are remarkably susceptible to *Streptococcus pneumoniae* infection and are protected by reconstitution with isolated pure human CRP, or by anti-pneumococcal antibodies. Autologous mouse CRP is evidently essential for innate resistance to pneumococcal infection before antibodies are produced. Our findings are consistent with the significant association between clinical pneumococcal infection and non-coding human *CRP* gene polymorphisms which affect CRP expression. Deficiency or loss of function variation in CRP may therefore be lethal at the first early-life encounter with this ubiquitous virulent pathogen, explaining the invariant presence and structure of CRP in human adults.

## Introduction

The physiological functions of human C-reactive protein (CRP), named for its interaction with pneumococcal somatic C-polysaccharide, in which it specifically binds the phosphocholine residues,^1^ are not known because no deficiency or even structural variants of the protein have been described. There are some extremely rare coding polymorphisms of the *CRP* gene but the variant proteins that they encode have not yet been reported. Such strict structural conservation suggests that CRP may have a function that is important for survival, potentially in innate immunity. Injection of human CRP into mice at the time of inoculation with virulent pneumococci confers efficient protection against sepsis^2^–^4^ but administration of human CRP after inoculation of the bacteria does not protect. Indeed, all patients with active pneumococcal infections have greatly increased plasma CRP concentrations and abundant circulating human CRP so it evidently does not control established pneumococcal sepsis.

The gene coding and amino acid sequences, homopentameric molecular assembly and calcium-dependent binding of CRP to phosphocholine residues are all phylogenetically conserved,^5^ for example mouse and human CRP share 71% amino acid sequence identity. But baseline plasma concentration, acute-phase behaviour, ligand precipitation, agglutination and complement fixation vary widely between the CRP of even closely related species.^5^ Hence, functional observations across species or effects of human CRP in mice cannot necessarily be reliably extrapolated to humans, and the *in vivo* role of autologous CRP in host defence has not previously been studied directly. We therefore created pure-line *Crp* gene-deleted C57BL/6 mice using C57BL/6 embryonic stem (ES) cells and characterized both their spontaneous phenotype and their responses to various challenges relevant to suspected functions of CRP.

## Material and methods

### 

#### Gene deletion

Pure-line C57BL/6 *Crp* knockout mice were generated by gene targeting in C57BL/6 ES cells and breeding with C57BL/6 partners (see Supporting information, Fig. S1), obviating any backcrossing. The CRP coding sequence was precisely deleted along with the intron, and the selectable marker was removed by FLP recombination *in vivo*, using C57BL/6-Tg (CAG-Flpe) 2Arte mice transgenic for the FLP recombinase (TaconicArtemis, model no. 7089). In the resulting knockout allele, 891 bp of genomic sequence was replaced by a 48-bp fragment containing a single FRT site. All mice used for experiments were FLP^−^.

#### Protein isolation and assays

Human CRP and serum amyloid P component (SAP), > 99.9% pure and fully structurally and functionally intact, were isolated by calcium-dependent affinity chromatography followed by specific solid-phase absorption of contaminants and final gel filtration, as previously described.^6^,^7^ Murine CRP (Life Diagnostics Inc., West Chester, PA) and serum amyloid A protein (SAA; Tridelta Development Ltd, Maynooth, Co. Kildare, Ireland) were assayed by ELISA. Anti-nuclear antibody (ANA) was detected by immunofluorescence as described.^8^

#### Bacterial infection

*Streptococcus pneumoniae* isolates were from clinical pneumococcal infection cases or carriers, and from type cultures, and were typed, cultured and quantified by standard methods. Mouse infection studies were conducted as previously described^9^ in sex-matched and closely age-matched groups of adult *Crp* knockout and wild-type control C57BL/6 mice, and were humanely killed at 72 hr.

#### Study approval

All mouse experiments were fully compliant with UK Home Office regulations, approved by the UCL Institutional Review Board.

## Results

### Spontaneous phenotype of CRP-deficient mice

**Figure 1:**
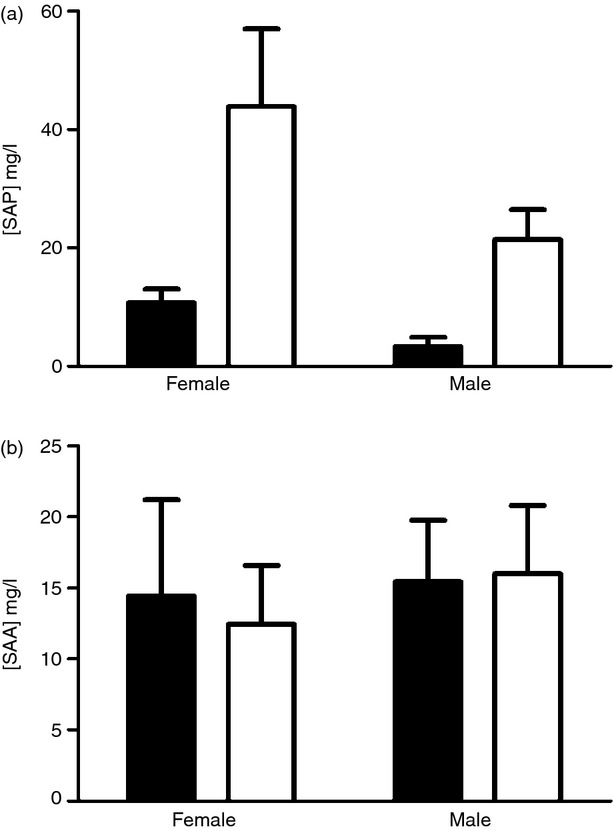
Baseline concentrations of acute-phase proteins in sex and age matched *Crp* knockout and control wild-type C57BL/6 mice. Mean (SD), *n* = 7 per group, for (a) serum amyloid P component (SAP) and (b) serum amyloid A protein (SAA).

Homozygous *Crp* gene-deleted C57BL/6 mice developed normally, were healthy and fertile, as previously independently reported by Teupser *et al*.^10^ in their own strain of C57BL/6 *Crp* knockout mice. No mouse CRP was detectable in the serum of our knockouts whereas the baseline concentration in adult wild-type C57BL/6 mice was 5–9 mg/l. At 24–48 hr after subcutaneous injection of 0.2 ml 2% weight/volume aqueous silver nitrate, a strong inflammatory stimulus, the circulating mouse CRP concentration rose to a peak of 17 mg/l. Mean (SD) body weights at weaning of pooled equal numbers of male and female mice were: wild-type 10.2 (1.95) g, *n* = 26; *Crp* knockout 9.0 (2.76) g, *n* = 28, *P* = 0.0819 by Mann–Whitney *U*-test; at 7 weeks, wild-type 19.5 (2.41), *n* = 19; *Crp* knockout 18.6 (2.17), *n* = 19, *P* = 0.265 by Student's *t*-test; at 10 weeks, wild-type 21.4 (2.13), *n* = 20; *Crp* knockout 21.6 (2.03), *n* = 18, *P* = 0.7025 by Student's *t*-test. Adult males weighed more than adult females at 7 and 10 weeks but there was no significant difference between genotypes. Lifespan was normal, with survival to 1100 days by Kaplan–Meier analysis, *n* = 87 wild-type and 120 *Crp* knockouts, *P* = 0.1768. Serum biochemistry (see Supporting information, Fig. S2) and haematological parameters were not significantly different from wild-type C57BL/6 mice. The baseline serum concentration of mouse SAP, which is a major murine acute-phase reactant,^11^ was very slightly higher in the *Crp* knockout mice than in wild-type controls (Fig. 1a), consistent with modestly up-regulated transcription of the *Sap* gene, which is immediately adjacent and very closely related to *Crp*, and/or increased efficiency of SAP protein secretion. However, the serum concentrations of SAA, the other sensitive murine acute-phase reactant, were not increased (Fig. 1b), excluding an underlying pro-inflammatory state in the *Crp* knockouts.

### Non-infectious challenges

**Figure 2:**
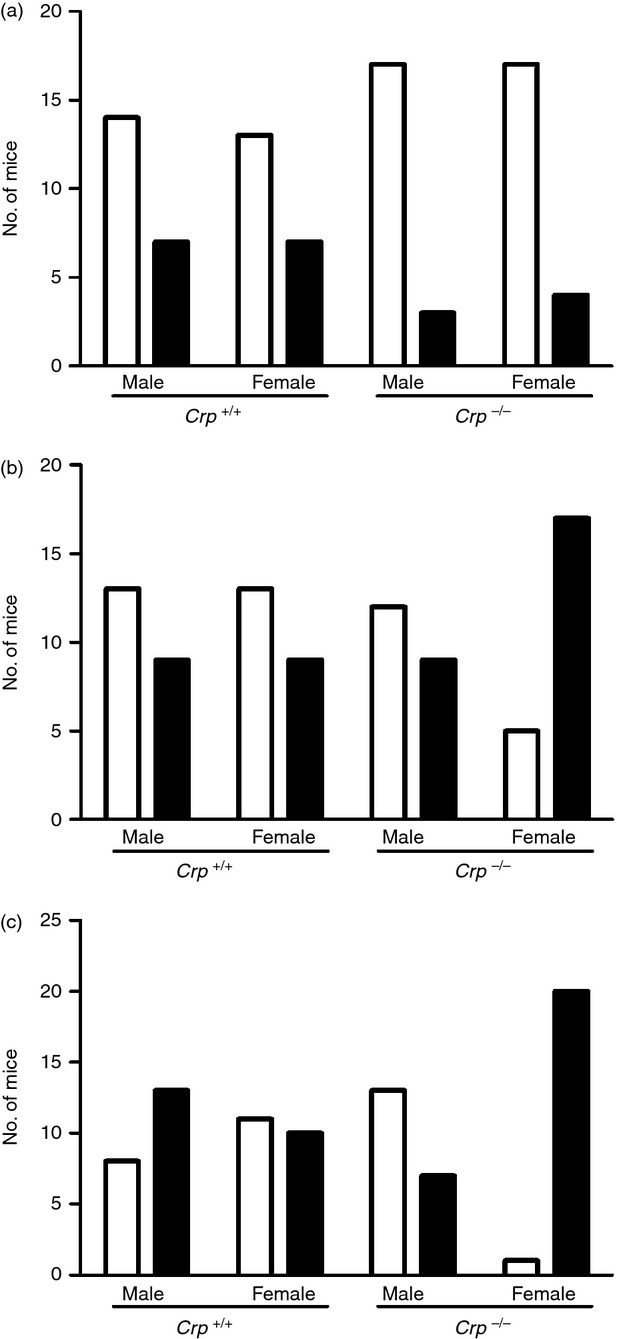
Spontaneous anti-nuclear antibody (ANA) production in sex-matched and age-matched *Crp* knockout and control wild-type C57BL/6 mice. Open columns, ANA-negative mice with titre < 1/80; solid columns, ANA-positive mice with titre > 1/80. (a) 3 months of age; (b) 6 months; (c) 9 months. Female *Crp* knockout mice had significantly more ANA by Fisher's exact test at 9 months (*P* = 0.03) and at 12 months (*P* = 0.002).

C-reactive protein may have a role in preventing ANA formation^12^–^14^ and spontaneous ANA production became significantly greater among female but not male *Crp* knockouts at 9 months of age (% mice with ANA positive at 1 : 80 serum dilution, *n* = 22 per group, *P* = 0.03 by Fisher's exact test) and 12 months of age (*P* = 0.002, *n* = 21) (Fig. 2). A *Crp* transgenic study is required to determine whether this modest effect is indeed due to CRP deficiency because the *sle1* locus, which controls ANA production, is adjacent to the *Crp* gene on distal mouse chromosome 1. However, the response to immunization with apoptotic thymocytes did not differ between wild-type and *Crp* knockout mice (not shown).

**Figure 3:**
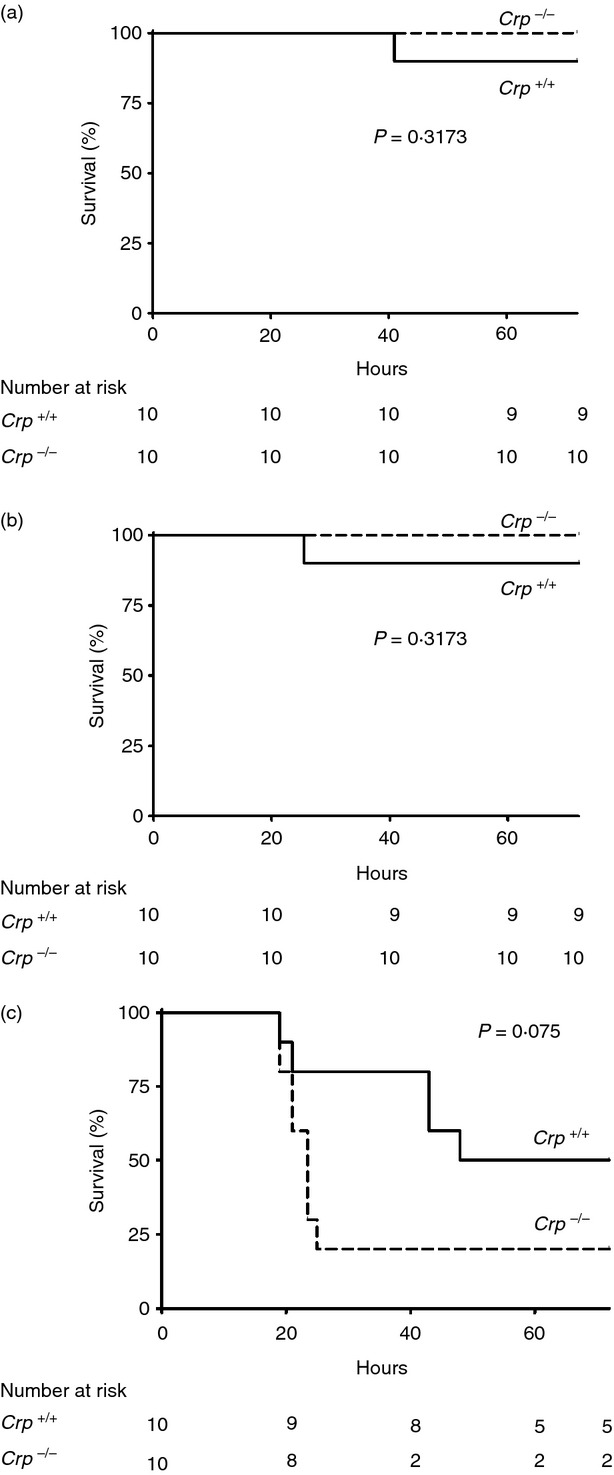
Lipopolysaccharide (LPS) lethality in sex-matched and age-matched *Crp* knockout and control wild-type C57BL/6 mice. There was no significant difference between survival of mice in the two groups after administration of LPS at 10 mg/kg (a), 20 mg/kg (b) or 40 mg/kg (c). The apparent trend to greater susceptibility in the *Crp* knockout mice receiving 40 mg/kg, which did not achieve statistical significance, is well within the typical variation in outcome after administration of LPS to mice. Biological significance is not supported by the fact that the knockouts were not more susceptible than wild-type mice to smaller doses of LPS.

Consistent with our previous finding that transgenic human CRP expression did not protect mice against the toxicity of Gram-negative bacterial lipopolysaccharide,^15^ there was no significant difference between *Crp* knockouts and wild-type mice in weight loss and clinical score or lethality (Fig. 3) produced by intraperitoneal injection of endotoxin at 10, 20 or 40 mg/kg. Local Shwartzman reactions induced by intradermal injection of endotoxin from *Salmonella typhimurium*, or from *Haemophilus influenzae* strains with and without phosphocholine expression, also did not differ between wild-type and *Crp* knockouts.

### Pneumococcal infection

**Figure 4:**
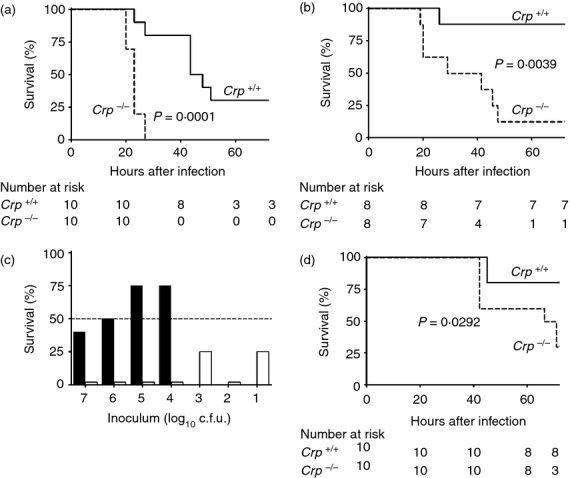
Susceptibility to infection with *Streptococcus pneumoniae*. Kaplan–Meier survival analysis up to 72 hr after intraperitoneal inoculation of *Crp* knockout mice and wild-type littermate controls with 10^5^ colony-forming units (c.f.u.) per mouse of virulent *S. pneumoniae* type 6B, isolated from two different clinical cases of pneumococcal pneumonia, (a), isolate Tz7712-2 and (b) isolate RF200. The results are representative of three independent experiments. (c) Lethality at 72 hr of infection by intraperitoneal inoculation of different doses of virulent *S. pneumoniae* type 6B isolate Tz7712-2 in *Crp* knockout mice (open bars) and wild-type littermate controls (closed bars) (three to five mice per group). Wild-type mice were not tested with the lowest doses because they survived larger doses. For clarity, 0% survival is indicated by bars of 2%. The results are representative of four independent experiments with doses of 10^3^, 10^4^ and 10^5^ c.f.u.; for ethical reasons of animal welfare, doses of 10^1^, 10^2^, 10^6^ and 10^7^ c.f.u. were tested only in the experiment shown, as the results of further experiments clearly would not provide further information. (d) Kaplan–Meier survival analysis up to 72 hr after intraperitoneal inoculation of *Crp* knockout mice and wild-type littermate controls with 5 × 10^4^ c.f.u. per mouse of *S. pneumoniae* type 27 (strain HO 8452 0293) (single experiment). All *P* values are from log-rank tests.

C-reactive protein-deficient mice were dramatically more susceptible than age- and sex-matched wild-type C57BL/6 controls to lethal infection after intraperitoneal inoculation of two independent clinical isolates of virulent type 6B *S. pneumoniae* (strains Tz7712-2 and RF200) (Fig. 4a,b), with LD_50_ values of < 10 and about 10^6^ organisms, respectively (Fig. 4c). Lethality corresponded exactly with viable bacterial counts in the blood at 20 hr after inoculation; only mice with < 10^4^ colony-forming units per ml survived at 72 hr. The *Crp* knockout mice were also much more susceptible to infection with type 27 *S. pneumoniae* which, unlike type 6B and most other pneumococci, have phosphocholine in the capsule^16^ (Fig.4d). However, CRP-deficient mice were not more susceptible to infection with pneumococcal strains RF206, serotype 1, and RF32, serotype 19F, mucoid colonies of heavily capsulated organisms isolated from blood cultures; or strain Tz1003-2, serotype 6A, isolated from a carrier.

**Figure 5:**
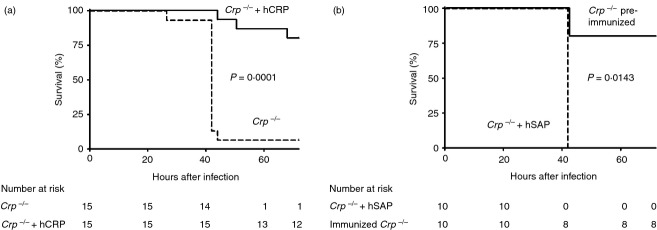
Protection, by human C-reactive protein (hCRP) or previous immunization, of *Crp* knockout mice against lethal infection with *Streptococcus pneumoniae*. (a) *Crp* knockout mice received either 3.5 μg of isolated highly purified human CRP per mouse, or vehicle alone, immediately before intraperitoneal inoculation of 10^3^ colony-forming units (c.f.u.) per mouse of virulent *S. pneumoniae* type 6B (isolate Tz7712-2). The results are representative of two independent experiments. (b) *Crp* knockout mice received 1 mg of isolated highly purified human serum amyloid P component (hSAP) per mouse immediately before intraperitoneal inoculation with 10^3^ c.f.u. per mouse of virulent *S. pneumoniae* type 6B (isolate Tz7712-2), while another group of *Crp* knockouts, which received the same inoculum at the same time, had been immunized 4 weeks previously by intramuscular injection of 9 × 10^7^ heat-killed *S. pneumoniae* type 6B (isolate Tz7712-2) (single experiment). Kaplan–Meier survival curves; *P*-values are from log-rank tests.

Intraperitoneal injection of just 3.5 μg of isolated pure human CRP immediately before inoculation of *Crp* knockouts with 10^3^ colony-forming units of type 6B *S. pneumoniae*, enabled survival at 72 hr of 80% (12/15) of the animals, compared with only 7% (1/15) among vehicle-only controls (*P* = 0.0001 by Fisher's exact test) (Fig. 5a), confirming that susceptibility is indeed due to CRP deficiency. The very low abundance of mouse CRP has so far prevented the isolation of sufficient intact protein for use in reconstitution studies. Importantly, human SAP, which is structurally very similar to human CRP but does not bind to phosphocholine or C-polysaccharide under physiological solvent conditions,^17^ conferred no protection. In contrast, mice that had been immunized with dead pneumococci 4 weeks before the live challenge were strongly protected (Fig. 5b), consistent with the optimal protection provided by the T15 germ-line idiotype anti-phosphocholine antibody.^18^ Autologous mouse CRP is therefore required for innate resistance to pneumococcal infection before sufficient specific antibodies are produced, but can be replaced by isolated human CRP or a previous anti-pneumococcal immune response.

## Discussion

The pathogenic and host resistance mechanisms in pneumococcal infection are controversial^19^,^20^ and complicated by the variety and variability of pneumococci and by differences between humans and experimental animals. Pneumococcal phosphocholine expression is crucial for respiratory tract colonization and for the virulence of invasive infection.^19^,^20^ However, *in vivo* protection by autologous CRP, the ubiquitous circulating phosphocholine-binding protein, has not previously been studied. The dramatically increased susceptibility of *Crp* knockout mice to types 6B and 27 pneumococci is therefore remarkable.

Despite universal binding to free phosphocholine, there are significant differences between the CRP of different species in recognition of macromolecules such as C-polysaccharide and phospholipids in which the phosphocholine residues must be accessible and in the correct stereochemistry. Native, non-aggregated human CRP does not bind to the ubiquitous phosphocholine head groups in the plasma membranes of healthy living cells, on native low-density lipoprotein particles or on pure phosphatidyl choline liposomes, but it avidly binds to membranes of dead or damaged cells, modified low-density lipoproteins or liposomes containing lysophosphatidyl choline.^21^–^23^ Even subtle differences in phosphocholine recognition affect host resistance to pneumococci: antibody clones arising during maturation of the anti-phosphocholine response, which bind more avidly to *p*-nitrophenyl phosphocholine or aminophenyl-phosphocholine than to phosphocholine itself, are not protective.^24^ Furthermore, pneumococcal surface protein A, the choline-binding bacterial protein which is a major pneumococcal virulence factor, competes with CRP for binding to bacterial phosphocholine.^25^ Insufficient accessibility of phosphocholine for binding by CRP therefore reduces or abolishes the protective role of CRP *in vivo* and this is presumably why we found that *Crp* knockout mice were not more susceptible to infection with strains 1, 6A and 19F. Similarly, in our preliminary experiments, CRP-deficient mice were not more susceptible to *Trypanosoma brucei*, *Plasmodium berghei*, *Leishmania donovani* and *Brugia malayi* (see Supporting information, Fig. S3), despite the universal presence of phosphocholine in these parasites.^26^ None of them are natural pathogens for mice and their phosphocholine residues may not be accessible to, or recognized by, mouse CRP. Also, regardless of the level of phosphocholine expression by *Haemophilus influenzae* strains, we could not establish infections with this organism in either wild-type or *Crp* knockout mice.

Complement-dependent neutrophil phagocytosis and killing of bacteria are essential for host defence against pneumococci and are required for optimal protection of mice by human CRP.^19^,^20^ C3 activation by pneumococci in CRP-deficient mouse serum and its modest dose-dependent enhancement by human CRP have been reported.^25^ However, our type 6B organisms activated C3 in both *Crp* knockout and wild-type mouse serum. Hence, while complement is necessary for optimal opsonophagocytosis and bacterial killing, CRP may have other effects in host defence. In particular, potent agglutination of bacteria by CRP may alone be sufficient to reduce spread *in vivo*, enabling more efficient neutrophil phagocytosis and killing without having to invoke the involvement of Fc receptors, cytokine production or dendritic cells, all of which have previously been suggested.^20^ A key role for CRP in clumping the first invading or inoculated bacteria is also consistent with human CRP only protecting mice when given at the time of infection and not thereafter, and with the failure of increased CRP production to control established clinical pneumococcal infection in patients.

*Streptococcus pneumoniae* is not a natural pathogen for mice and the conclusion, based on mouse CRP deficiency, that human CRP has a necessary role in human host defence against pneumococci must be cautious. However, there is a convincing association between increased incidence of pneumococcal sepsis and non-coding variants of the human *CRP* gene, which cause lower baseline CRP values.^27^–^29^ Upper respiratory tract carriage of pneumococci is very common in humans and these bacteria are extremely virulent when they enter the tissues, so a critical role of human CRP in innate resistance is a compelling explanation for the absence of any deficiency or even structural variation in CRP. It is also consistent with the high frequency of anti-phosphocholine idiotypes in the germ-line antibody repertoire. Absence of human CRP or loss of function variants may be lethal defects for neonates or infants who encounter virulent phosphocholine-bearing pneumococci when not adequately protected by maternal or their own anti-phosphocholine antibodies.

## Abbreviations

ANA: anti-nuclear antibody
CRP: C-reactive protein
*Crp*: mouse C-reactive protein gene
ES: embryonic stem
SAA: serum amyloid A protein
SAP: serum amyloid P component

## Supporting Information

**Figure S1.** Generation of C57BL/6 *Crp* knockout mice by gene targeting in C57BL/6 ES cells and breeding with C57BL/6 partners.

**Figure S2.** Serum biochemistry in wild-type and *Crp* knockout C57BL/6 mice. Mean (SD), *n* = 8–24.

**Figure S3.** *Crp* knockouts were not more susceptible then wild-type C57BL/6 mice to parasitic infections.
