# Supplementary figures and images for: C-reactive protein is essential for innate resistance to pneumococcal infection
Source: Immunology. 2014 Jun 10;142(3):414–20. doi: 10.1111/imm.12266 (PMC4080957; doi:10.1111/imm.12266)

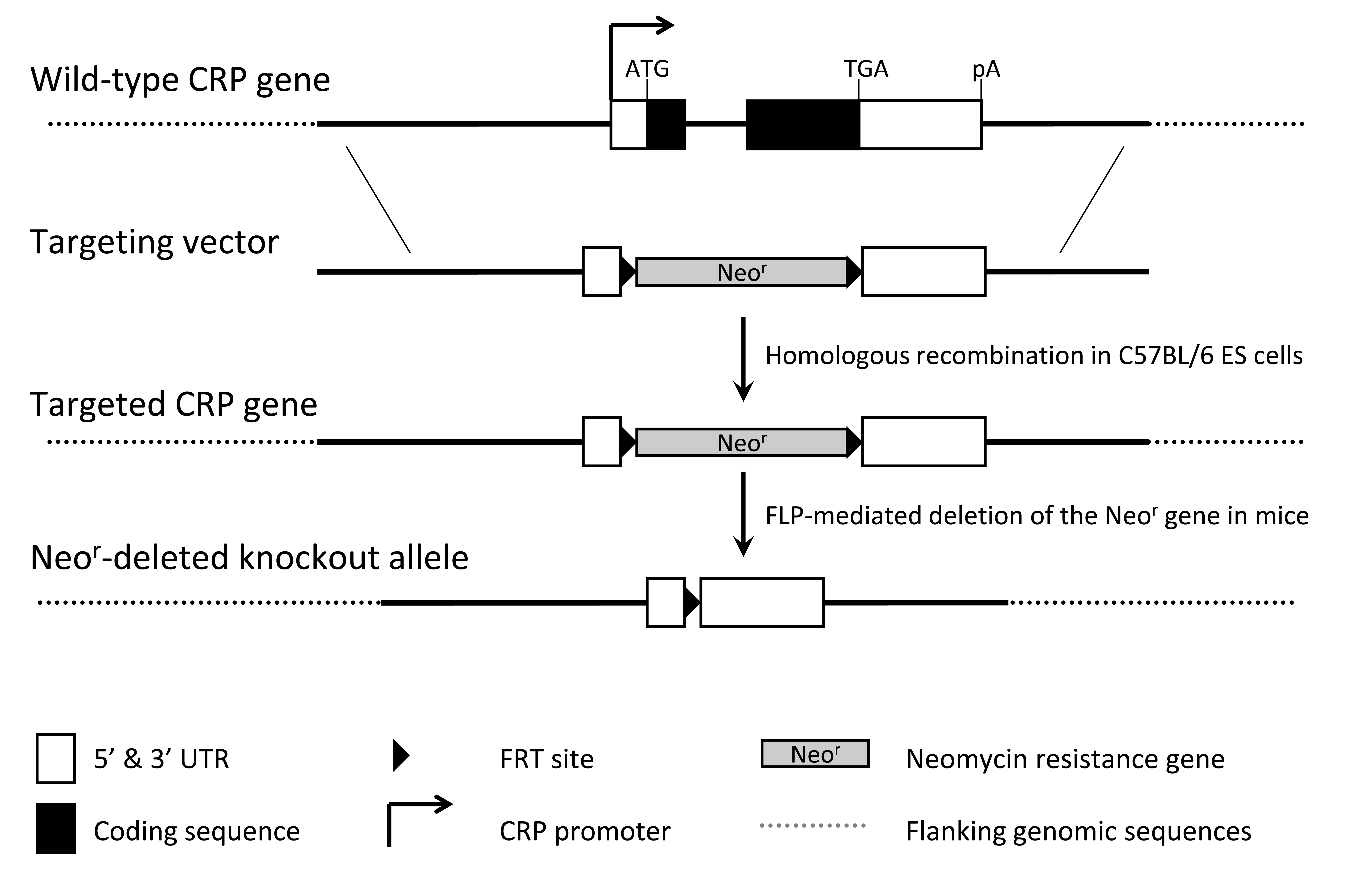

Supplement: Supplementary file 1 [file imm0142-0414-sd1.tif]
