# Supplementary material for: C-reactive protein is essential for innate resistance to pneumococcal infection
Source: Immunology. 2014 Jun 10;142(3):414–20. doi: 10.1111/imm.12266 (PMC4080957; doi:10.1111/imm.12266)
Supplement: Supplementary file 4 [file imm0142-0414-sd4.docx]

SUPPLEMENTARY FIGURES FOR:

**C‑reactive Protein is Essential for Innate Resistance to Pneumococcal Infection**

**J. Paul Simons,^1^ Jutta M. Loeffler,^1^ Raya Al**‑**Shawi,^1^ Stephan Ellmerich,^1^ Winston L. Hutchinson,^1^ Glenys A. Tennent,^1^ Aviva Petrie,^2^ John G. Raynes,^3^ J. Brian de Souza,^3^ Rachel A. Lawrence,^4^ Kevin D. Read^5^ and Mark B. Pepys^1^**

^1^Wolfson Drug Discovery Unit, Centre for Amyloidosis and Acute Phase Proteins, University College London, London, ^2^Biostatistics Unit, UCL Eastman Dental Institute, London, ^3^Department of Immunology and Infection, London School of Hygiene and Tropical Medicine, London, ^4^Department of Comparative Biomedical Sciences, Royal Veterinary College, London, and ^5^Drug Discovery Unit, Division of Biological Chemistry and Drug Discovery, University of Dundee, Dundee, UK

**Legends to Figures**

**Figure S1**Generation of C57BL/6 *Crp* knockout mice by gene targeting in C57BL/6 ES cells and breeding with C57BL/6 partners. The targeting vector was 10kb of genomic DNA with an FRT‑flanked Neo^r^ cassette inserted to precisely replace the CRP coding sequence and the intron. After Southern blot confirmation of the structure of the 5’ and 3’ flanks of the targeted allele, *Crp*‑deleted ES cells were used to generate chimaeras which were bred with C57BL/6-Tg(CAG-Flpe)2Arte mice transgenic for the FLP recombinase (TaconicArtemis, model no. 7089) to remove the selection cassette. Deletion of the Neo^r^ cassette was confirmed in FLP transgenic mice that carried the targeted *Crp* allele, comprising 891bp of genomic sequence replaced by a 48bp fragment containing a single FRT site. The targeted *Crp*^‑^ allele was then bred away from the FLP transgene in subsequent generations and heterozygous *Crp*^+/‑^ mice were bred to generate *Crp*^‑/‑^ and *Crp*^+/+^ homozygotes which were maintained as pure breeding lines.

**Figure S2**Serum biochemistry in wild type and *Crp* knockout C57BL/6 mice. Mean (SD), *n* = 8-24. There were no significant differences between wild type (filled bars) and *Crp* knockout mice (open bars) for any analyte.

**Figure S3***Crp* knockouts were not more susceptible then wild type C57BL/6 mice to parasitic infections.  All animal procedures were performed in accordance with the Animals (Scientific Procedures) Act 1986 and Specified Animal Pathogens Order 1998**.** Infected animals had access to food and water *ad libitum* and were housed under a 12 hour light/dark photoperiod. Malaria (A, B).  *Plasmodium berghei* ANKA parasites from liquid nitrogen stocks were subjected to one *in vivo* passage prior to use in experimental infection. Mice were infected intravenously with 10^4^ parasitized red blood cells and parasitaemia was estimated on Giemsa stained blood films from day 3 onwards. Neurological symptoms were closely monitored from day 5. Trypanosomes (C).  Cryopreserved *Trypanosoma brucei brucei* (s427) stabilate, 1 ml in 10% glycerol, was diluted with Hanks Balanced Salt Solution containing 20 mM glucose to give 5 x 10^3^ trypomastigotes/ml. Female mice were intraperitoneally injected with 0.2 ml/animal (~1 x 10^3^ trypomastigotes) on day 0 and parasitaemia was monitored daily in tail blood smears, counting 20 fields at x400 magnification. If more than 50 parasites/field were seen, a formal hemocytometer count was performed. Mice with a parasite burden over 1 x 10^8^/ml were promptly and humanely killed. Leishmania (D)**.**  Female mice, 10 wild type and 10 *Crp* knockouts, were inoculated intravenously on day 0 with hamster spleen-derived stibogluconate sensitive *Leishmania donovani* (LV9 strain, WHO designation MHOM/ET/67/L82) amastigotes, 0.2 ml/animal (equivalent to ~1.5 - 2 x 10^7^ amastigotes) (Wyllie and Fairlamb, 2006). Two mice from each group were killed on days 7, 14, 21, 28 and 35 after inoculation and their livers removed and weighed. Parasite load was determined by microscopic examination of Giemsa stained liver smears, counting the number of amastigotes/500 liver cells at x1000 magnification, and expressing the parasite load in Leishman-Donovan units, mean amastigotes/liver cell x mg liver (Bradley and Kirkley, 1977). Filaria (E).  *Brugia malayi* infected gerbils (*Meriones unguiculatus*) were obtained from TRS Labs (Athens, Georgia, USA). Gerbils were infected by intraperitoneal injection of 400 *B. malayi* L3. Fluid recovered from peritoneal lavage with RPMI-1640 100-300 days post infection was centrifuged over lymphocyte separation medium (MP Biomedicals, USA) to separate microfilariae from host granulocytes. *Crp* knockout mice and their wild type counterparts were infected with 200,000 microfilariae per mouse by intravenous injection and microfilaria survival was determined 28 d post-infection in 200 µl of heparinised cardiac blood.

Bradley DJ, Kirkley J. Regulation of *Leishmania* populations within the host. I. the variable course of *Leishmania donovani* infections in mice. Clin Exp Immunol 1977; **30:**119-29.

Wyllie S, Fairlamb AH. Refinement of techniques for the propagation of *Leishmania donovani* in hamsters. Acta Trop 2006; **97:**364-9.
